# Mother-to-child transmission of HIV and its predictors among HIV-exposed infants at Bamenda Regional Hospital, Cameroon

**DOI:** 10.4102/ajlm.v6i1.589

**Published:** 2017-12-14

**Authors:** Victor N. Fondoh, Njong A. Mom

**Affiliations:** 1 Bamenda Regional Hospital Laboratory, Bamenda, North-West Region, Cameroon; 2 Faculty of Economics and Management Sciences, University of Bamenda, Bamenda, North-West Cameroon

## Abstract

**Background:**

Mother-to-child transmission (MTCT) of HIV, has been a major global public health burden. Despite the use of antiretroviral prophylaxis by HIV-positive pregnant women and their infants, safe obstetric practice and safe infant feeding habits in the prevention of MTCT of HIV, the prevalence of HIV among HIV-exposed infants is still high in Cameroon.

**Objective:**

Our objectives were to determine the prevalence, assess the predictors and determine the effect of combination antiretroviral therapy (cART) on MTCT of HIV at the regional hospital in Bamenda, Cameroon.

**Methods:**

This was a retrospective study. Secondary data from 877 HIV-exposed infants aged ≤ 72 weeks were extracted from the records between January 2008 and December 2014. The predictors and effect of cART on MTCT of HIV were analysed using a multivariable logistic regression model and risk analysis, respectively.

**Results:**

Out of 877 HIV-exposed infants, 62 were positive for HIV, giving a prevalence of 7.1%. Maternal antiretroviral intervention and infant age group were statistically significant predictors of MTCT of HIV. HIV-positive mothers who were on cART were 2.49 times less likely to transmit HIV than those who were not on cART.

**Conclusion:**

In order to reduce the prevalence of HIV among HIV-exposed infants, maternal antiretroviral intervention should be targeted and the use of cART by HIV-positive pregnant women should be encouraged.

## Introduction

Mother-to-child transmission (MTCT) of HIV refers to vertical transmission of HIV from an HIV-positive mother to her baby at one or more of the following stages: pregnancy, labour, delivery or breastfeeding.^1,2^ Globally, this accounts for 90% of HIV infections in children under the age of 15 years.^3^ In 2016, 90% of the estimated four million children living with HIV resided in sub-Saharan Africa.^4^ It is also estimated that 5% – 10% of MTCT occurs during pregnancy, 10% – 20% during labour and delivery and 10% – 20% through breastfeeding. The risk of transmission is 15% – 45% without intervention. With intervention, the risk is reduced to 5% in breastfeeding populations^5^ and less than 2% in non-breastfeeding populations.^3^ One-third of HIV-positive children die within one year of birth and half before their second birthday without intervention.^6,7^ In 2012, Cameroon registered an 8.4% HIV prevalence in HIV-exposed infants with the north-west region registering 3.7%.^8^

Interventions for the prevention of mother to child transmission (PMTCT) of HIV include: antiretroviral prophylaxis given to women during pregnancy and labour and to their infants within the first weeks of life, safe obstetric practices and safe infant feeding habits.^9,11^ These interventions were being implemented globally by the PMTCT programme initiated by the World Health Organization in order to meet goal four of the Millennium Development Goals which centers on the reduction of child mortality.

The implementation of the PMTCT programme was started at the Regional Hospital Bamenda (RHB), Cameroon, in 2008 by the Elizabeth Glaser Pediatric AIDS Foundation of the Cameroon Baptist Convention Health Board. This programme is being funded by the United States President’s Emergency Plan for AIDS Relief.^12^ There are many reports on the outcomes of the PMTCT interventions.^13,14^ Our objectives were to determine the prevalence of MTCT of HIV among HIV-exposed infants in Cameroon, assess its predictors and determine the efficacy of combination antiretroviral therapy (cART) as an intervention.

## Methodology

### Ethical considerations

Authorisation to conduct this research was obtained from the Catholic University of Cameroon’s institutional review board (No. 020/HEPM/CATUC-IRB/15). Approval to carry out the research at RHB was obtained from the RHB Institutional Review Board (No. 001/APP/RDPH/RHB/IRB). Authorisation to collect data was obtained from the RHB Paediatric Treatment and Care Centre (RHB-PTCC) Head of Department. Consent for the HIV-exposed infants enrolled for follow-up at the RHB-PTCC was given by their mother or caregiver orally.

### Study area

The study site was the RHB, situated in Bamenda, in the north-west region of Cameroon. The RHB was chosen because it is a level-two hospital for five district hospitals serving a population of 302 749 inhabitants. It also has one of the largest ante-natal clinics that implements the PMTCT programme, an extended programme on immunisation centre for referral of HIV-exposed infants for follow-up and the RHB-PTCC, where HIV-exposed infants are enrolled and followed up.

### Research design

This was a retrospective study. Secondary data from routine follow-up records of HIV-exposed infants were reviewed and collected from the RHB-PTCC from the period January 2008 to December 2014. Follow-up of HIV-positive mothers and their infants was based on the World Health Organization guidelines that evolved over the duration of the study period in response to changes in international guidelines and newly available information.^15^

During care at the ante-natal clinic, pregnant women enrolled in the PMTCT programme were tested for HIV by serology using Determine HIV-1/2 Ag/Ab Combo (Alere Medical Co., Ltd, Matsuhidai, Matsudo-Shi, Chiba; first line) and ImmunoComb II HIV 1&2 BiSpot (Orgenics Ltd, Yavne, Israel; second line) kits. Pregnant women with no test records and those who had never known their HIV status were tested at the maternity ward upon presentation for delivery. Those who were HIV-positive were placed on antiretroviral therapy during pregnancy, labour and delivery depending on when their status was identified. The antiretroviral drugs were usually used singly or as cART, depending on availability and/or clinical conditions. The medications used were: nevirapine, zidovudine, lamivudine, tenofovir, efavirenz, abacavir, lopinavir/ritonavir, atazanavir/ritonavir. The cART could be one of the following regimens: zidovudine + lamivudine + nevirapine, zidovudine + lamivudine + efavirenz, abacavir + lamivudine + efavirenz, tenofovir + lamivudine + efavirenz, tenofovir + lamivudine + lopinavir/ritonavir, tenofovir + lamivudine + atazanavir/ritonavir. The women were also counseled on feeding interventions for their infants. HIV-exposed infants were placed on nevirapine or zidovudine, (depending on availability, immediately) after birth at the maternity ward and as scheduled by the RHB-PTCC.^15^

The HIV status of the infants were determined at six weeks of age or as early as possible after enrolment in the centre. A heel-prick dried blood spot was collected from the infants at the RHB laboratory and sent to the Early Infant Diagnosis Laboratory in Mutengene (from 2008 to 2011) or the Tuberculosis Reference Laboratory, Bamenda (from 2012 to 2014), all in Cameroon, for qualitative analysis of HIV DNA by polymerase chain reaction (PCR) testing. Infants were declared positive for HIV if the HIV DNA PCR test was positive.

#### Inclusion criteria

Included in the studies were HIV-exposed infants up to 72 weeks old, who had HIV results (including that of their respective mothers) registered in the HIV DNA PCR register and whose mothers gave consent (orally).

#### Exclusion criteria

Excluded from the studies were infants who came in for a second HIV DNA PCR test, were older than 72 weeks of age, had no test results (including that of their respective mothers) registered in the HIV DNA PCR register, and those whose mothers did not consent (orally).

### Data collection

Data collection was conducted by three trained reviewers. The following records were reviewed: RHB-PTCC results register, PMTCT register and follow-up forms of each registered infant. The data from each participant were cross-checked for consistency in the three registers. Detailed information on the records and management of the infants was obtained from key informants (health care providers) working at the RHB-PTCC. Data were collected using a structured data collection format. Data were coded and entered into a Microsoft Excel 2010 spreadsheet (Microsoft Corporation, Redmond, Washington, United States).

The variables were selected based on the PMTCT of HIV interventions (methods) that directly affect the physiology of the mother and child. The dependent variable was the outcome of interest: HIV transmission (the HIV status of exposed infant). The independent variables were infant age at HIV diagnosis (0–6 weeks or > 6–72 weeks), sex (male or female), maternal antiretroviral intervention (on cART or not on cART), obstetric intervention (vaginal or caesarean section), infant antiretroviral intervention at time of diagnosis (nevirapine or zidovudine) and the infant feeding intervention (breastfed or formula) at time of diagnosis. Data were double-checked by a second reviewer in order to guarantee quality. Mothers of twins were counted twice.

### Data analysis

Analyses were conducted using R statistical software (R, version 3.3.3; R Foundation for Statistical Computing, Vienna, Austria). Descriptive statistics were done using frequency distribution. Univariable analysis was used to determine the association between the PMTCT interventions and HIV transmission. The predictors of MTCT of HIV were assessed by building a multivariable logistic regression model, adding the variables in a forward selection process in order, starting with those with the smallest *p*-value from the univariable analysis. Variables with a *p*-value greater than or equal to 0.2 on the univariable analysis were not included in the multivariable analysis. The final model was based on the selection of the smallest Akaike information criterion,^14,16,17^ and risk analysis was used to determine the effect of cART. Analyses were conducted using 95% confidence intervals, and a *p*-value of less than 0.05 was considered statistically significant.

## Results

**FIGURE 1:**
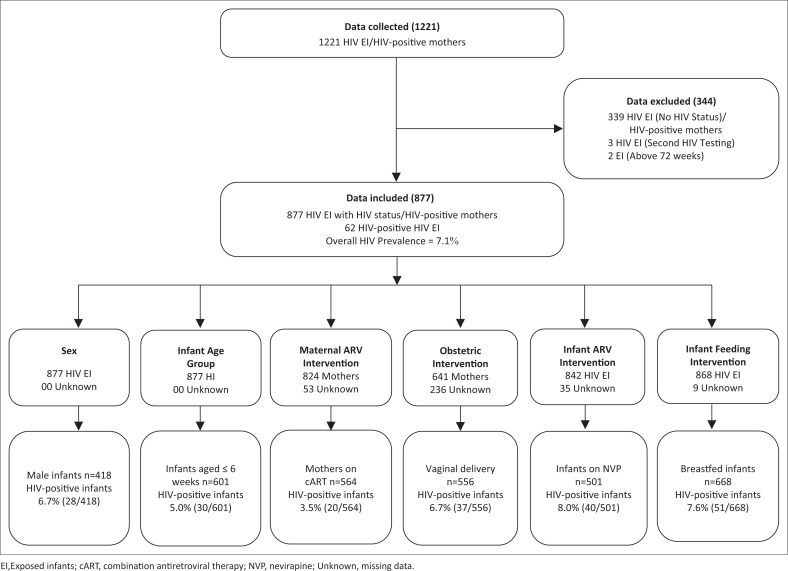
Flow Chart of representation of the population used to estimate the Prevalence of HIV in HIV exposed infants in the Regional Hospital Bamenda.

**FIGURE 2:**
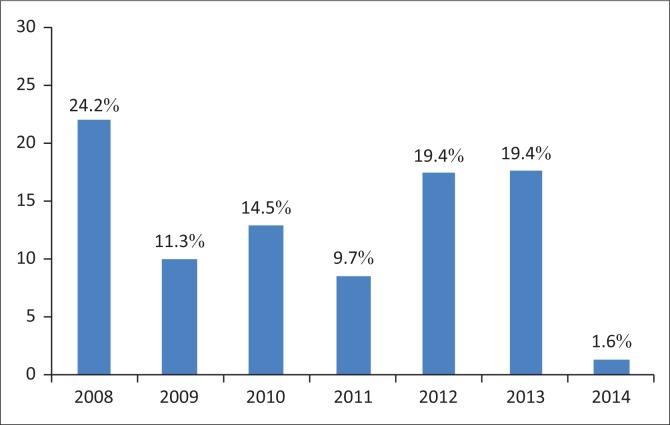
Prevalence of HIV in HIV-exposed infants over the years 2008–2014.

Data from 1221 HIV-exposed infants (including 1221 of their respective HIV-positive mothers) were collected between January 2008 and December 2014 (Figure 1). A total of 877 HIV-exposed infants (including 877 of their respective HIV-positive mothers) with HIV results were included in the analysis, and 344 were excluded (three infants came in for a second HIV testing; two infants were older than 72 weeks and 339 infants did not have complete data for HIV status). Of the 877 HIV-exposed infants, 62 were HIV-positive giving an overall HIV prevalence of 7.1% among HIV-exposed infants (Figure 2).

Of the 877 HIV-exposed infants included in the analysis, 418 (47.7%) were male, of whom 28 (6.7%) were HIV-positive (Table 1). The median age at HIV diagnosis was 6 weeks (range = 2–48 weeks), and 601 infants (69.0%) were in the age group 0–6 weeks, of which 30 (5.0%) were positive for HIV. Of the 877 HIV-positive mothers, data for 824 were analysed for maternal antiretroviral intervention; 53 had incomplete data and were excluded (Figure 1). Twenty (3.5%) of the 564 mothers on cART were HIV-positive. Of the 877 HIV-exposed infants, data for 641 were analysed for infant obstetric intervention, and 236 with incomplete data were excluded. Thirty-seven (6.7%) of the 556 infants delivered vaginally were HIV-positive. Of the 877 HIV-exposed infants, data for 842 were analysed for infant antiretroviral intervention, and 35 with incomplete data were excluded. Forty (8.0%) of the 501 infants on nevirapine were HIV-positive. Of the 877 HIV-exposed infants, data for 868 were analysed for infant feeding intervention, and nine with incomplete data were excluded. Fifty-one (7.6%) of the 668 infants who were breastfed were HIV-positive.

**TABLE 1:** HIV transmission among HIV-exposed infants – Univariable analysis

Variable	N†	n‡ (%)	Chi-square value	*p*-value
**Infant sex**			0.167	0.683
Male	418	28 (6.7)		
Female	459	34 (7.4)		
**Infant age group (weeks)**			12.550	0.001*
0–6	601	30 (5.0)		
> 6–72	276	32 (11.6)		
**Maternal ARV intervention**			15.473	< 0.001*
cART	564	20 (3.5)		
Not cART	260	27 (9.8)		
**Obstetric intervention**			0.019	0.890
Vaginal	556	37 (6.7)		
Caesarean	85	6 (7.1)		
**Infant ARV intervention**			1.891	0.089
NVP	501	40 (8.0)		
ZDV	341	17 (4.9)		
**Infant feeding intervention**			1.635	0.201
Breastfed	668	51 (7.6)		
Formula fed	200	10 (5.0)		

* Statistically significant value.

Abbreviations: ARV, antiretroviral; cART, combination antiretroviral therapy; NVP, nevirapine; ZDV, zidovudine.

† Number of HIV-exposed infants tested for HIV.

‡ Number of HIV-positive HIV-exposed infants.

Our univariable analysis indicated that there were statistically significant associations between HIV transmission and both age group (chi-square: 12.550, *p* = 0.001) and maternal antiretroviral intervention (chi square: 15.473, *p* < 0.001) (Table 2). There were no statistically significant associations between HIV transmission and infant sex (chi-square: 0.167, *p* = 0.683), infant feeding intervention (chi-square: 1.635, *p* = 0.201), infant antiretroviral intervention (chi-square: 1.891, *p* = 0.089) or obstetric intervention (chi-square: 0.019, *p* = 0.890).

**TABLE 2:** Multivariable logistic regression of predictors of HIV transmission among HIV-exposed infants

Variable	Unadjusted OR		Adjusted OR	
	OR (95%CI)	*p*-value†	AOR (95%CI)	*p*-value†
**Infant sex**				
Male	1.00	0.683		
Female	0.90 (0.53–1.51)			
**Infant age group (weeks)**				
0–6	1.00	< 0.001*		0.017*
> 6–72	2.50 (1.48–4.20)		2.34 (1.61–4.72)	
**Infant feeding intervention**				
Breastfed	1.00	0.201		
Formula fed	0.64 (0.32–1.28)			
**Maternal ARV intervention**				
cART	1.00	< 0.001*		0.001*
Not cART	3.15 (1.73–5.73)		2.49 (1.23–5.02)	
**Infant ARV intervention**				
ZDV	1.00	0.091		
NVP	1.65 (0.92–2.97)			
**Obstetric intervention**				
Vaginal	1.00	0.890		0.814**
Caesarean	1.07 (0.44–2.61)		1.13 (1.16–4.72)	

* , Statistically significant at *p* < 0.05.

** Non-statistically significant from the multivariable logistic regression analysis.

Abbreviations: AOR, adjusted odd ratio; ARV, antiretroviral CI, confidence interval; cART, combination antiretroviral therapy; OR, odd ratio; NVP, nevirapine; ZDV, zidovudine.

† *p*-values were obtained by chi-square test.

Following our multi-variable logistic regression model, obstetric intervention, although not statistically significant, was retained as it improved the overall fit of the model based on the Akaike information criterion. Infant sex, infant feeding intervention and infant antiretroviral intervention did not improve the overall fit of the model and thus were excluded. The final model included three variables: maternal antiretroviral intervention, infant age group and obstetric intervention. Based on this model, maternal antiretroviral intervention (*p* = 0.001) and infant age group (*p* = 0.017) were statistically significant predictors of MTCT of HIV (Table 2). Our risk analysis revealed that mothers on cART were less likely to transmit HIV to their infants (adjusted odds ratio (OR) = 2.49, 95% confidence interval (CI): 1.23–5.02) compared to those who were not on cART. Similarly, infants aged 0–6 weeks at the time of HIV diagnosis were less likely to be infected with HIV compared to those aged > 6–72 weeks (adjusted OR = 2.34, 95% CI: 1.61–4.72).

## Discussion

Our study revealed that the prevalence of HIV among HIV-exposed infants in Bamenda, Cameroon, was 7.1%, which is still a burden to society. This may be due to non-adherence to antiretroviral protocols by mothers and infants, vaginal delivery or breastfeeding.^10,18,19^ A similar result (7.0%) was reported from Nigeria between 2011 and 2012.^20^

Lower prevalence was reported in south India (6.5% in 2010)^21^, South Africa (5.8% between 2004 and 2008^23^, 5.4% between 2008 and 2010^22^) and in high-income countries (2.9% between 2001 and 2003 in Europe^16^, less than 2% in United States, Europe, Brazil and Bahamas between 1997 and 2000^11^). This may be due to the universal use of cART, elective caesarean sections and avoidance of breastfeeding in developed countries^24^. Such preventive approaches are limited in poor countries (including Cameroon) due to poor funding and social and cultural norms.^24^

Higher prevalence was reported in Zambia (12.2% between 2007 and 2010)^26^ and Nigeria (9.1% in 2010).^27^ This might suggest better management of the PMTCT programme at our study site. The north-west region registered a prevalence of 3.7% in 2012^8^, far lower than the prevalence of 7.1% in our study. The high prevalence might be due to the fact that, RHB is a level-two referral hospital with a high influx of patients resulting from the availability of specialised and satisfactory services including (but not limited to) the laboratory, paediatric, obstetric and gynecological services).

Our multiple logistic regression model revealed that infant age at HIV diagnosis and maternal antiretroviral intervention were statistically significant predictors of MTCT of HIV. Our observations are similar to those observed in other models in Ethiopia and Europe, where infant age group at diagnosis and maternal antiretroviral intervention, respectively, were statistically significant predictors of MTCT of HIV.^13,16^

In our study, infants aged > 6– 72 weeks were at higher risk of HIV transmission compared to those aged 0–6 weeks. This might be because most of the infants who were diagnosed at older than six weeks of age were those who were sick, coupled with the fact that some were not on antiretroviral drugs. Late diagnosis at the study site might have resulted from poor communication, stigmatisation and discrimination, poor training of health care providers and lack of stock of antiretroviral drugs.

Poor communication between the ante-natal clinic, delivery and postnatal facilities and lack of good information systems may lead to late diagnosis of HIV-exposed infants.^28,29^ At the RHB, there is no traceable standard system or network (software or internet) to link the ante-natal clinic, maternity, postnatal care, immunisation centre, the RHB-PTCC and the community. There is a communication link between the caregiver and the RHB-PTCC, but this link is established during enrolment at the centre. There may be some gaps in communication if an infant’s mother is critically ill or dies. Due to inadequate counselling and education during antenatal visits, some mothers are unsure of the type of tests or activities to be done on their infants at the early infant diagnosis services. Thus, they may not be motivated to take their child for follow-up treatment and care services.^30^

Fear of stigmatisation and discrimination, especially at the immunisation center (where some of these infants are identified) increases maternal reluctance to identify themselves for proper follow-up referral.^30,31,32^ During the vaccination process at the RHB vaccination center, the vaccinators identify the cards of HIV-exposed infants and invite them for a discussion after the process, which creates a stigma on the mothers. Secondly, the nature of the infrastructure may also contribute to stigmatisation. All infants identified for dried blood spot collection are referred to the blood bank at the RHB laboratory. Babies brought to the blood bank are assumed to be an HIV-exposed child, which is a stigma.

We found that HIV-positive women who were not on cART were 2.49 times more likely to transmit HIV to their babies compared to those who were on cART. All the mothers in our study who were not on cART were either on nevirapine or zidovudine. Nevirapine and zidovudine are single-drug regimens, which are not as effective as cART. However, this justifies the assertion that cART decreases the risk of MTCT of HIV^33^ and is more effective than nevirapine or zidovudine.^34^ Several studies have reported viral resistance to nevirapine in pregnant women, including women in Cameroon.^35^

Poor training of health care provides, stock-outs of cART or delays by some HIV-positive mothers to start cART may be other reasons for the high risk of MTCT of HIV. This may be caused by administrative bottlenecks or poor management at some or all levels. A report from Cameroon in 2012 indicated that there was inadequate training of health care providers, stock-out of antiretroviral drugs, poor antenatal coverage, fewer pregnant women going for HIV testing, low coverage of HIV-positive women on antiretroviral drugs, weak follow-up of HIV-exposed infants and fewer HIV-exposed infants tested at six weeks of age, which led to ineffective implementation of the PMTCT programme.^8,36^

The risk of HIV transmission by caesarean section delivery in this study was 2.34 times higher than vaginal delivery. Some of the caesarean sections were elective and some were a result of complication during delivery, but all were counted as caesarean section. This might have accounted for the high risk of MTCT of HIV through caesarean section deliveries that resulted due to complications during labour, including membrane rupture before the caesarean section was done and subsequent blood contact with HIV-contaminated blood.^37^

### Recommendations

In order to reduce the spread of HIV, particular attention should be directed to the infant age group at diagnosis and maternal antiretroviral intervention. All pregnant HIV-positive women in Cameroon should be placed on cART. Antiretroviral drugs reduce maternal viral load and risk of HIV transmission to the baby and partner.^38^ These recommendations can only be achieved through a joint endeavour by the government, the institutions, the communities and individuals.

The government should provide resources including continuous supply of cART to all pregnant women, improve the diagnostic services for maternal and infant diagnosis, subsidise delivery kits, train and employ more health caregivers and improve on infrastructure. A ‘one-stop shop’ infrastructure with network and a Web-based medical record system to link the ante-natal clinic to the maternity ward, postnatal, immunisation centre, the RHB-PTCC, the laboratory and the community should be created at the RHB to better manage the HIV-exposed infants, improve confidentiality and reduce stigmatisation.

The institutions should improve on the protocols of treatment and encourage HIV-positive pregnant women to take cART during pregnancy in order to keep their baby safe, ensure HIV DNA PCR testing for infants at six weeks, encourage vaginal delivery (discourage the use of caesarean section except when it is unavoidable) and encourage formula feeding of HIV-exposed infants.

The community should avoid stigmatisation of HIV-positive individuals. HIV-positive mothers and HIV-exposed babies should adhere to their routines: take their drugs regularly and report any unusual development in their systems timeously, accept their identity and come out of stigmatisation.

### Limitations

Since we used secondary data from the records of the RHB-PTCC, it was difficult to control the inconsistencies of missing data from one or more of the parameters. Although missing data did not affect our results since it was considered during analysis, it could have increased our sample size for better interpretation and reduction of the risk of bias. Since this was not a clinical trial, it was difficult to have a clear-cut distinction between infants who were exclusively breastfed or formula-fed because other food supplements or drugs including traditional medicine might have been given to the infants. We could not distinguish between those who had elective caesarean sections from those who had caesarean sections due to complications in pregnancy. Thus all were grouped as caesarean section. Since all the mothers and infants were said to be on antiretroviral drugs, it was not possible to distinctively assess those who had never been on antiretroviral drugs. We could not distinguish the specific regimen taken by the pregnant women on cART. Thus we considered all as cART. We also could not also distinguish those who were on a specific regimen because of availability or their clinical condition. The data for all variables were not available. This study was to assess the predictors of the PMTCT interventions and these predictors were available.

### Conclusion

Our study concludes that the prevalence of HIV among HIV-exposed infants was 7.1%. Infant age group at diagnosis and maternal antiretroviral intervention were statistically significant predictors of MTCT of HIV. The risk of MTCT of HIV when HIV-positive mothers were on cART was 2.49 times lower compared to those who were not on cART.
